# The complete chloroplast genome provides insight into the evolution and polymorphism of *Panax ginseng*

**DOI:** 10.3389/fpls.2014.00696

**Published:** 2015-01-14

**Authors:** Yongbing Zhao, Jinlong Yin, Haiyan Guo, Yuyu Zhang, Wen Xiao, Chen Sun, Jiayan Wu, Xiaobo Qu, Jun Yu, Xumin Wang, Jingfa Xiao

**Affiliations:** ^1^CAS Key Laboratory of Genome Sciences and Information, Beijing Institute of Genomics, Chinese Academy of SciencesBeijing, China; ^2^University of Chinese Academy of SciencesBeijing, China; ^3^School of Pharmaceutical Sciences, Changchun University of Chinese MedicineChangchun, China

**Keywords:** comparative genomics, SNP, minor allele, chloroplast genome, *Panax ginseng*

## Abstract

*Panax ginseng* C.A. Meyer (*P. ginseng*) is an important medicinal plant and is often used in traditional Chinese medicine. With next generation sequencing (NGS) technology, we determined the complete chloroplast genome sequences for four Chinese *P. ginseng* strains, which are Damaya (DMY), Ermaya (EMY), Gaolishen (GLS), and Yeshanshen (YSS). The total chloroplast genome sequence length for DMY, EMY, and GLS was 156,354 bp, while that for YSS was 156,355 bp. Comparative genomic analysis of the chloroplast genome sequences indicate that gene content, GC content, and gene order in DMY are quite similar to its relative species, and nucleotide sequence diversity of inverted repeat region (IR) is lower than that of its counterparts, large single copy region (LSC) and small single copy region (SSC). A comparison among these four *P. ginseng* strains revealed that the chloroplast genome sequences of DMY, EMY, and GLS were identical and YSS had a 1-bp insertion at base 5472. To further study the heterogeneity in chloroplast genome during domestication, high-resolution reads were mapped to the genome sequences to investigate the differences at the minor allele level; 208 minor allele sites with minor allele frequencies (MAF) of ≥0.05 were identified. The polymorphism site numbers per kb of chloroplast genome sequence for DMY, EMY, GLS, and YSS were 0.74, 0.59, 0.97, and 1.23, respectively. All the minor allele sites located in LSC and IR regions, and the four strains showed the same variation types (substitution base or indel) at all identified polymorphism sites. Comparison results of heterogeneity in the chloroplast genome sequences showed that the minor allele sites on the chloroplast genome were undergoing purifying selection to adapt to changing environment during domestication process. A study of *P. ginseng* chloroplast genome with particular focus on minor allele sites would aid in investigating the dynamics on the chloroplast genomes and different *P. ginseng* strains typing.

## Introduction

The chloroplast, an important plastid, plays an essential role in plant cell functions, including photosynthesis and carbon fixation (Neuhaus and Emes, 2000). In angiosperms, the chloroplast has a conserved quadripartite structure composed of two copies of inverted repeat (IR), one large single copy (LSC), and one small single copy (SSC) (Palmer, 1985). Comparative analysis indicates that most chloroplast genomes are highly conserved at the gene level (Jansen et al., 2005). Compared with the nuclear genome, the chloroplast genome has many other advantages, such as haploid and maternal inheritance (Birky, 2001). Since decoding the first chloroplast genome sequence (*Nicotiana tabacum*, Shinozaki et al., 1986) in 1986, over 200 complete chloroplast genomes have been sequenced and deposited in NCBI Organelle Genome Resources database. With the development of DNA sequencing technology, the chloroplast genomes have been widely used for plant identification and resolving phylogenetic relationships (Jansen et al., 2007; Moore et al., 2007, 2010; Parks et al., 2009).

*Panax ginseng*, a member of Araliaceae family, is native to China, Korea, and Russia. For thousands of years, traditional Chinese medicine has relied on *P*. *ginseng* to restore and enhance human health, and *P*. *ginseng* has been used as a tonic, stimulant, and agent to foster fatigue and stress-resistance for more than 2000 years. More recently, the pharmaceutical effects of *P*. *ginseng* have been shown by a host of studies, and *P*. *ginseng* has become one of the most well-known medicinal plants worldwide (Pazyar et al., 2012; Zheng et al., 2012; Gao et al., 2013).

Traditional methods to identify wild ginseng from cultivars are based on phenotypic observations, while morphological characteristics are often affected by environmental and developmental factors. Particularly during the early developmental stages, the seeds and seedlings of different ginseng cultivars are extremely morphologically similar; rendering their differentiation is quite difficult and sometimes impossible. Furthermore, morphological characteristics cannot be used for screening large numbers of ginseng samples. Therefore, a simple method of DNA analysis, rather than the traditional authentication methods, is clearly desirable. Lee et al. studied the phylogeny of *Panax* with chloroplast *trnC*–*trnD* intergenic region sequences (Lee and Wen, 2004). In 2004, the first *P*. *ginseng* chloroplast genome sequence, *P*. *schinseng* Nees (156,318 bp, Genbank accession number: NC_006290), was reported by Kim et al. (Kim and Lee, 2004). The general features of *P*. *schinseng* Nees chloroplast genome and genome structure dynamics compared to other chloroplast genome sequences have been well described. However, the data were insufficient for studying the genome dynamics and evolution of *P*. *ginseng* chloroplast. Herein, we present four complete Chinese *P*. *ginseng* chloroplast genome sequences based on next generation sequencing (NGS) technology. Comparative analyses of the chloroplast genomes between *P*. *ginseng* strains and other plants were conducted. At the same time, high resolution of genome sequences has made it easy to investigate genetic variations in the chloroplast genome sequence that have occurred over the course of domestication.

## Materials and methods

### Ethics statement

All plant samples (four kinds of Chinese ginseng cultivars) in current study were collected from E-Mu ginseng experimental base (N 43°47′45.72″, E128°6′7.88″, 46 m above sea level), Changchun University of Chinese Medicine with permission. Ginseng in current research does not involve endangered or protected species.

### Plant DNA extraction and sequencing

Four *P*. *ginseng* samples, including three domestic ginseng strains (DMY, EMY, and GLS) and one wild ginseng strain (YSS), were all collected from E-Mu ginseng experimental base. Morphological differences among these four strains mainly focus on rhizome, lateral root and so on, and the detailed differences were introduced in Supplementary Table S1. After cleaning, the fresh roots were frozen in liquid nitrogen and stored at −80°C until further processing. Changchun University of Chinese Medicine confirmed the identification of the four ginseng samples through morphology. The total mixed genomic DNA was extracted from fresh roots by using a Plant Genomic DNA Kit (Tiangen Biotech Co., China) following the manufacturer's instructions.

For each sample, pair-end reads with different insertion lengths were sequenced by Illumina HiSeq2000 system. Additionally, DMY was also sequenced with Roche/454 GS-FLX (Titanium) pyrosequencing machine for single-end fragment reads. The detailed sequencing information for four strains was listed in Supplementary Table S2.

### Chloroplast genome assembly and minor allele analysis

All raw HiSeq reads of four ginseng strains were filtered by quality control protocol with the following five rules: (i) there is no “N” in the entire read, (ii) the percentage of bases with quality <10 is 0, (iii) the percentage of bases with quality <15 is ≤3%, (iv) the percentage of bases with quality <20 is ≤5%, and (v) the average quality of all bases is ≥30. Only high quality reads satisfying all five rules were used in genome assembly and SNP calling (high quality reads information were listed Supplementary Table S2).

For DMY, all reads from 454 GS FLX sequences were directly assembled using Newbler (overlapMinMatchLength = 40 and overlapMinMatchIdentity = 90). According to Zhang's assembly strategy (Zhang et al., 2011), we used BLAT (-minIdentity = 90) to search large contigs (length ≥ 1 kb) with the conserved chloroplast genes (both identity and coverage on gene nucleotide sequence are no less than 90%). Those large contigs, which contain two or more conserved chloroplast genes, were selected as seed contigs in bb.454contignet (Iorizzo et al., 2012) script (set –lowlimit to 10). The chloroplast contig graph was also drawn with bb.454contignet, and low coverage contig branches (possibly including the DNA transferred from chloroplast to mitochondrial or nuclear genomes) were excluded manually (Zhang et al., 2011). Subsequently, SSC, LSC, and IR were connected into a circle. High-quality pair-end reads from three different insertion length libraries were employed to verify the contig connections and whether each base on the circle genome was supported by the primary allele base by using BWA (Li and Durbin, 2010) with default parameters and SAMTools (-Q = 20, -*m* = 5 on mpileup) (Li et al., 2009).

For EMY, GLS, and YSS, high-quality pair-end reads with different insertion lengths were BLAT-searched (set –minIdentity to 70 in BLAT) against all 207 chloroplast genomes (206 were downloaded from NCBI FTP, the other one is DMY chloroplast genome, and the accession numbers were listed in Supplementary Table S3). The matched reads (coverage and identity on each reads are no less than 70%) were considered as chloroplast-related reads, and 150,000 pairs of high-quality and chloroplast-related pair-end reads were selected randomly for assembly from different insertion length libraries respectively. Moreover, Velvet (Zerbino and Birney, 2008) was employed to assemble the reads into scaffolds (hash_length values are set to 63, 63, and 69 in velveth for EMY, GLS, and YSS respectively). For each of the other three ginseng strains, the order of scaffolds was determined by mapping scaffolds to DMY chloroplast genome with BLAT program (set –minIdentity to 90 in BLAT), and gaps were filled by fishing reads from high-quality pair-end reads with Gapcloser and then manually checked. The verification method for the chloroplast genome sequences of EMY, GLS, and YSS were the same as that for DMY.

In minor allele identification section, all high quality pair-end reads with an inserting length of 500 bp from all four strains were mapped onto DMY chloroplast genome using BWA with default value. The program rmdup in SAMTools was employed to remove duplicated reads and SNP calling. Finally, only alleles with MAF ≥0.05 were retained as minor allele candidates (De et al., 2008; Skoglund and Jakobsson, 2011; Iorizzo et al., 2013).

### Chloroplast gene annotation

Protein-coding genes in ginseng chloroplast genome were annotated by DOGMA (set protein identity to 50, and hits number to 10) (Wyman et al., 2004); start and stop sites of these annotated protein-coding genes were corrected manually. rRNA was detected by aligning rRNA sequences from other chloroplast genomes to ginseng chloroplast genome sequence using BLAT with global coverage and identify ≥90%, and tRNA was identified by tRNAscan-SE (Lowe and Eddy, 1997) with default parameters. The gene map of ginseng DMY chloroplast was drawn by OGDRAW (Lohse et al., 2007).

### Identification of repeat sequences

All repeat sequences were identified using microsatellite identification tool (MISA, http://pgrc.ipk-gatersleben.de/misa/misa.html), and each repeat sequence was ≥10 bp. Repeat sequences whose repeating sequence units were arranged from 2–6 bp and repeated not less than three times were considered as SSRs. Repeat sequences with lengths ≥20 bp were considered as large repeat sequences.

### Phylogenetic analysis

A phylogeny constructed for ginseng and other plants from Asterids was based on the whole genome sequences of LSC, SSC, and IR regions, and 52 protein-coding gene sequences. As to protein-coding sequences, each gene was aligned using Clustal W with default parameters (Goujon et al., 2010), and the aligned sequences from all genomes were connected in order of their sequence. MAFFT (-maxiterate = 10) (Katoh and Toh, 2010) was used to align the sequences from LSC, SSC, and IR, and poor alignment regions were manually checked and adjusted. The phylogenetic trees were constructed using maximum likelihood (ML) algorithm in MEGA5 (Tamura et al., 2011) with default parameters, and *Spinacia oleracea* (Order: Caryophyllales) and *Vitis vinfera* (Clade: Rosid) were used as outgroups.

Phylogeny among the four newly sequenced ginseng chloroplasts and *P*. *schinseng* Nees chloroplast were constructed on the basis of the genome sequences and MAFs in the polymorphism sites. Because clear differences were observed between *P*. *schinseng* Nees chloroplast and the newly sequenced chloroplasts, *P*. *schinseng* Nees chloroplast was used as an outgroup directly. According to UPGMA algorithm, the evolutionary relationship among the four newly sequenced chloroplasts was calculated based on MAF of 208 polymorphism sites.

### Genome structure analysis and nucleotide diversity

When comparing CDS, introns, and intergenic regions in LSC, SSC, and IR regions between ginseng and the other three closely related chloroplast genomes, the entire genome sequences were divided into series of small fragments by those exons of protein-coding genes. The fragment flanked by the same exons or genes would be taken as orthologous fragment. Orthologous fragments between ginseng and the other three closely related chloroplast genomes were aligned respectively, and then aligned sequences were orderly connected together according their regions. Nucleotide diversity and Ka/Ks in the different regions were calculated using DnaSP (version 5.10) (Librado and Rozas, 2009) with default parameters. The Ka/Ks value for each single protein-coding gene was calculated using the KaKs_caculator (-m MA -c 11) (Zhang et al., 2006).

## Results and discussion

### General features of *P. ginseng* chloroplast genome

#### Genome sequencing and assembly

The length for the whole chloroplast genome sequences for DMY, EMY, and GLS was 156,354 bp, while that for YSS was 156,355 bp. In DMY chloroplast genome sequence, the total length for LSC, SSC, and IR regions were 86,129 bp, 18,077 bp, and 26,074 bp, respectively. The accession numbers in GenBank for the four chloroplast genomes are KC686331 (DMY), KC686332 (EMY), KC686333 (GLS), and KF431956 (YSS). The clean reads for these newly sequenced chloroplast genomes were deposited in NCBI, and the accession number for SRA datasets are SRR1251992 (DMY), SRR1252006 (EMY), SRR1252007 (GLS), and SRR1252008 (YSS).

#### Genome annotation

In the determined nucleotide sequences of the chloroplast genome, DMY, EMY, and GLS chloroplast genome sequences were identical. Compared with these three chloroplast genome sequences, YSS chloroplast genome had only one base-pair insertion at 5472th base, which is located in the intron of *rps16* in LSC region. Therefore, DMY chloroplast genome was used as the representative of these newly sequenced *P*. *ginseng* chloroplast genomes for further comparative analyses.

**Figure 1:**
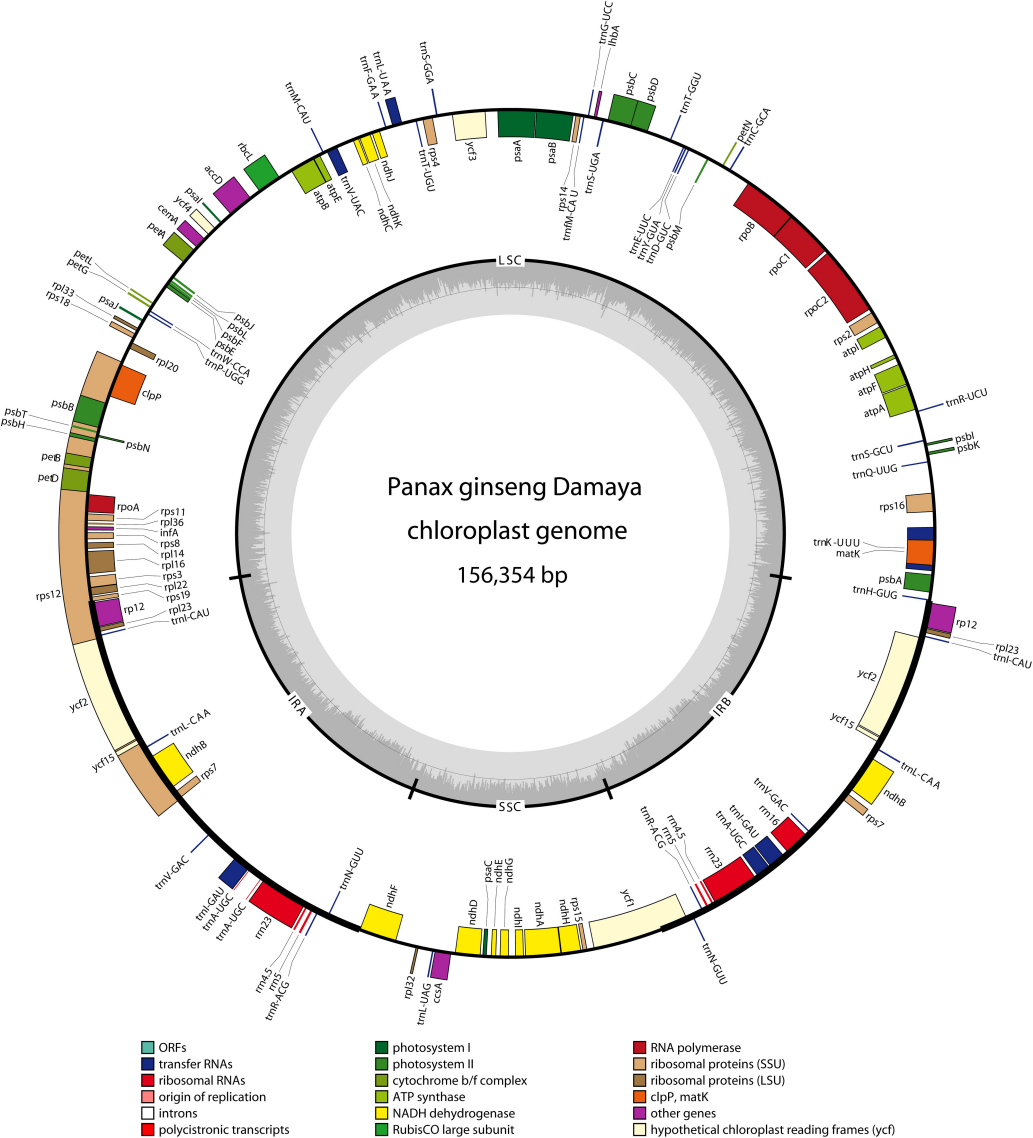
**Gene map of *Panax ginseng* DMY chloroplast genome sequence**. Genes shown outside the outer circle are transcribed clockwise, and those inside are transcribed counterclockwise. Genes belonging to different functional groups are color coded. The dashed area in the inner circle indicates GC content of the chloroplast.

In DMY chloroplast genome, 128 functional genes were identified, including 86 protein-coding genes, 34 tRNA genes, and 8 rRNA genes (Table 1), and the gene map for DMY chloroplast genome was shown as Figure 1. Among 86 protein-coding genes, 74 were single copy genes, 12 were duplicates, and 13 genes were composed of two or more exons. For instance, the *rps12* gene had three exons, which located in LSC region (one exon) and IR region (the other two). Among 42 RNA genes, 20 were unique and 22 were duplicates. 7 of 34 tRNA genes had one intron. A total of 26,162 codons were present in protein-coding genes of DMY chloroplast genome (Supplementary Table S4). Generally the nucleotide sequence of protein-coding gene begins with a start codon ATG, while there were some exceptions consist of the following: *rps19* began with GTG and *ndhD* began with ACG, which was also found in the chloroplast genomes of other plants, such as *Phoenix dactylifera* L (Yang et al., 2010); the *ycf15* gene in *Eleutherococcus senticosus* began with ATG (Yi et al., 2012), while that in *P*. *ginseng* began with GTG, with the first nucleotide changing from A to G.

**Table 1:** **Gene contents in *P*. *ginseng* DMY chloroplast genome**.

**Category for genes**	**Group of genes**	**Name of genes**
Self-replication	rRNA genes	*rrn16*(×*2*), *rrn23*(×*2*), *rrn4.5*(×*2*), *rrn5*(×*2*)
	tRNA genes	34 trn genes(7 contain an intron, 12 in the IR regions)
		trnA-UGC*(×*2*), trnC-GCA, trnD-GUC, trnE-UUC, trnF-GAA, trnG-UCC, trnH-GUG, trnI-GAU*(×*2*), trnK-UUU*, *trnL-CAA*(×*2*), trnL-UAA*, *trnL-UAG, trnM-CAU*(×*2*), *trnN-GUU*(×*2*), *trnP-UGG, trnQ-UUG, trnR-ACG*(×*2*), *trnR-UCU, trnS-GCU, trnS-GGA, trnS-UGA, trnT-GGU, trnT-UGU, trnV-GAC*(×*2*), trnV-UAC*, *trnW-CCA, trnY-GUA*
	Small subunit of ribosome	*rps2, rps3, rps4, rps7*(×*2*), rps8, rps11, rps12**, rps14, rps15, rps16*, *rps18, rps19*
	Large subunit of ribosome	rpl2*(×*2*), rpl14, rpl16*, *rpl20, rpl22, rpl23*(×*2*),*rpl32, rpl33, rpl36*
	DNA dependent RNA polymerase	rpoA, rpoB, rpoC1*, *rpoC2*
Genes for photosynthesis	Subunits of NADH-dehydrogenase	ndhA*, ndhB*(×*2*), *ndhC, ndhD, ndhE, ndhF, ndhG, ndhH, ndhI, ndhJ, ndhK*
	Subunits of photosystem I	psaA, psaB, psaC, psaI, psaJ, ycf3**
	Subunits of photosystem II	*psbA, psbB, psbC, psbD, psbE, psbF, psbH, psbI, psbJ, psbK, psbL, psbM, psbN, psbT*
	Subunits of cytochrome b/f complex	petA, petB*, petD*, *petG, petL, petN*
	Subunits of ATP synthase	atpA, atpB, atpE, atpF*, *atpH, atpI*
	Large subunit of rubisco	*rbcL*
Other genes	Translational initiation factor	*infA*
	Maturase	*matK*
	Protease	clpP**
	Envelope membrane protein	*cemA*
	Subunit of Acetyl-CoA-carboxylase	*accD*
	c-type cytochrome synthesis gene	*ccsA*
Genes of unknown function	Open Reading Frames (ORF, ycf)	*ycf1, ycf2*(×*2*), *ycf4, ycf15*(×2), *lhbA*

*One and two asterisks after gene names reflect one- and two-intron containing genes, respectively. Genes located in IR regions are indicated by the (×2) symbol after the gene name. The rps12 gene is divided: the 5 '-rps12 is located in LSC region and the 3 '-rps12 in IR region*.

#### Simple sequence repeats in DMY chloroplast genome

Simple sequence repeats (SSRs), which are also known as microsatellites and short tandem repeats (STRs), present high diversity in copy numbers. SSRs are important genetic molecular markers for population genetics (Doorduin et al., 2011; He et al., 2012) and are widely used for plant typing (Xue et al., 2012; Yang et al., 2011). With the microsatellite identification tool (MISA), 30 SSRs were detected in *P*. *ginseng* DMY chloroplast genome (Table 2), including 18 homopolymers, 1 dipolymer, 8 tetrapolymers, 2 pentapolymers, and 1 hexapolymer. In 19 homopolymers and dipolymers, 15 SSRs were only composed of A or T bases. In the other 11 SSRs, more than half of the bases were composed of A or T bases. Therefore, SSRs in DMY chloroplasts are AT-rich. Among these SSRs, 10 were located in intergenic regions, 2 in rRNA gene (*rrn23*), and 8 in protein-coding genes (*rpoA, rpoB, rpoC2, atpB, psbM*, and 3 in *ycf1*). SSRs with the length of repeat unit not less than two bases were not identified in the previously reported *P*. *schinseng* Nees chloroplast genome (Kim and Lee, 2004). Comparing with *P*. *ginseng* DMY chloroplast genome, copy number variations in *P*. *schinseng* Nees chloroplast genome have introduced some difference in homopolymer SSR. (A)13 (4823–4835 bp) and (G)11 (105,431–105,441 bp) in *P*. *ginseng* DMY chloroplast genome were designated as (A)12 (4822–4833 bp) and (G)10 (105,406–105,415 bp) in *P*. *schinseng* Nees chloroplast genome, which was caused by the deletions in *P*. *schinseng* Nees chloroplast genome.

**Table 2:** **Simple sequence repeats in *P*. *ginseng* DMY chloroplast genome**.

**Unit**	**Length**	**No. SSRs**	**Position on genome**
A	10	1	17677–17686
	11	1	23946–23956
	13	2	4823–4835, 14249–14261
C	10	2	7503–7512, 38191-38200
	11	1	137043–137053
G	11	1	105431–105441
T	10	7	27594–27603*(rpoB)*, 56528–56537*(atpB)*, 71553–71562, 80110–80119*(rpoA)*, 83153–83162, 127890–127899*(ycf1)*, 130063–130072*(ycf1)*
	11	3	19889–19899*(rpoC2)*, 83064–83074, 128582–128592*(ycf1)*
TA	14	1	85868–85881
AAGA	12	1	30782–30793
TCTT	12	1	30804–30815
AATT	12	1	30948–30959*(psbM)*
ATTT	12	1	34090–34101
TATT	12	1	69890–69901
AAAG	12	1	72233–72244
AGGT	12	1	107514–107525*(rrn23)*
CTAC	12	1	134957–134968*(rrn23)*
ATTAG	15	1	100769–100783
CTAAT	15	1	141701–141715
CATAGT	18	1	74295–74312

*Gene name in the bracket indicates that this SSR located on the gene*.

In addition to SSR sequences, repeats with lengths ≥20 bp were designated as large repeat sequences in DMY chloroplast, and 5 large repeat sequences were detected (Table 3). Two of them were located in intergenic regions. The other three were located in protein-coding regions, one in the *ycf1* gene and two in the *ycf2* gene. Because *ycf2* is located in IR region, each *ycf2* is comprised of one large repeat sequence only, which has also been reported in *Sesamum indicum* chloroplast genome (Yi and Kim, 2012).

**Table 3:** **Long repeat sequences in *P*. *ginseng* DMY chloroplast genome**.

**Repeat pattern**	**Size (bp)**	**Position**	**Location**
(CTACATC)3	21	1945–1965	Intergenic region
(CGATATTGATGCTAGTGA)4	72	92801–92872	*ycf2*
(ATATCGTCACTAGCATCA)4	72	149606–149677	*ycf2*
(AGAAACCCCAACAACGGAAGAAAGGGGGGAAAGTGAGGAAGAAACAGATGTAGAAAT)4	228	111304–111531	Intergenic region
(GTTTCTATTTCTACATCTGTTTCTTCCTCACTTTCCCCCCTTTCTTCCGTTGTTGGG)4	228	130947–131174	*ycf1*

### Comparative genomics and phylogenetic analyses across species

#### Phylogenetic analysis of P. ginseng chloroplast genome

**Figure 2:**
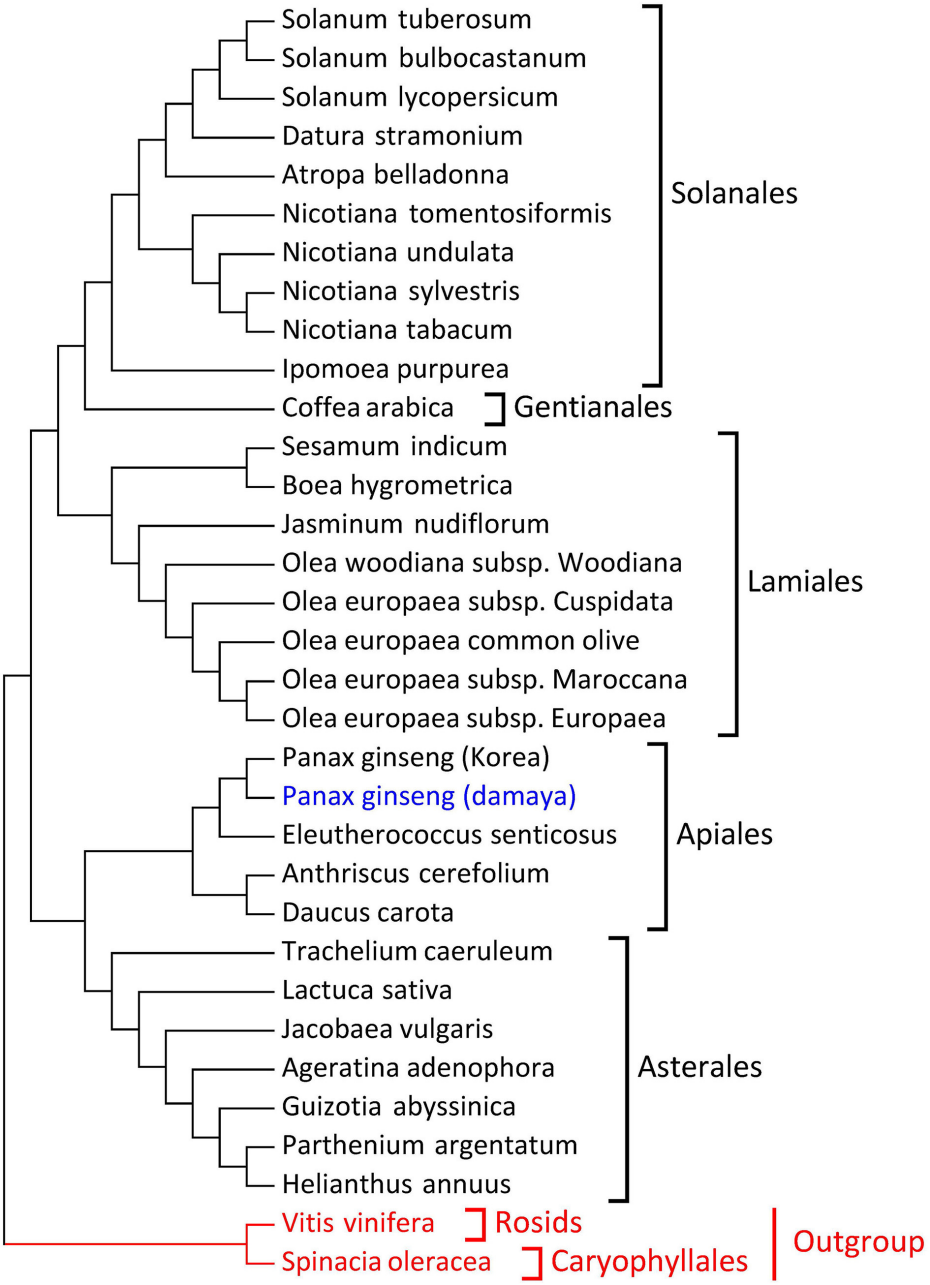
**Phylogenetic tree based on 52 protein-coding genes (maximum likelihood)**. DMY is marked in blue and the two outgroup species are marked in red.

To study the phylogenetic relationships of ginseng, 31 plants from Asterids clade were employed, and *Spinacia oleracea* and *Vitis vinifera* were incorporated as the outgroups (Supplementary Table S5). LSC, SSC, IR, and protein-coding regions were employed to construct the phylogenetic tree respectively. As to protein-coding region, 55 conserved protein-coding genes were employed (Supplementary Table S6), and the results were shown in Figure 2. The results for LSC, SSC, and IR regions were presented in Supplementary Figure S1A–1C. The phylogenetic relationship from LSC, SSC, and protein-coding regions were almost identical, and the topological relation also agreed with the taxonomy of core eudicots (Figure 2 and Supplementary Figure S1). The phylogenetic relationships based on IR region showed high similarities with the LSC, SSC and protein-coding regions' results, while *Ipomoea purpurea* from Solanales and *Trachelium caeruleum* from Asterales did not located in their original branches as their taxonomy but clustered into a new branch, which may be caused by frequent recombination and expansion/contraction in IR region (Wicke et al., 2011). Among these four phylogenetic trees, *P*. *ginseng* DMY and other plants in Apiales, including *P*. *schinseng* Nees, *Eleutherococcus senticosus* (*E*. *senticosus*), *Anthriscus cerefolium* (*A*. *cerefolium*), and *Daucus carota* (*D*. *carota*), had stable, topological relationships. It is apparent that the conserved protein-coding genes of the plant chloroplast genome can adequately trace the phylogenetic relationships of core eudicot plants, which have been proven by a series of other works (Wu et al., 2007; Lin et al., 2010; Liu et al., 2013; Qian et al., 2013). Meanwhile, the LSC and SSC sequences of chloroplasts could also be used for phylogenetic analyses of core eudicot plants.

#### Dynamics in IR region of P. ginseng chloroplast genome

**Figure 3:**
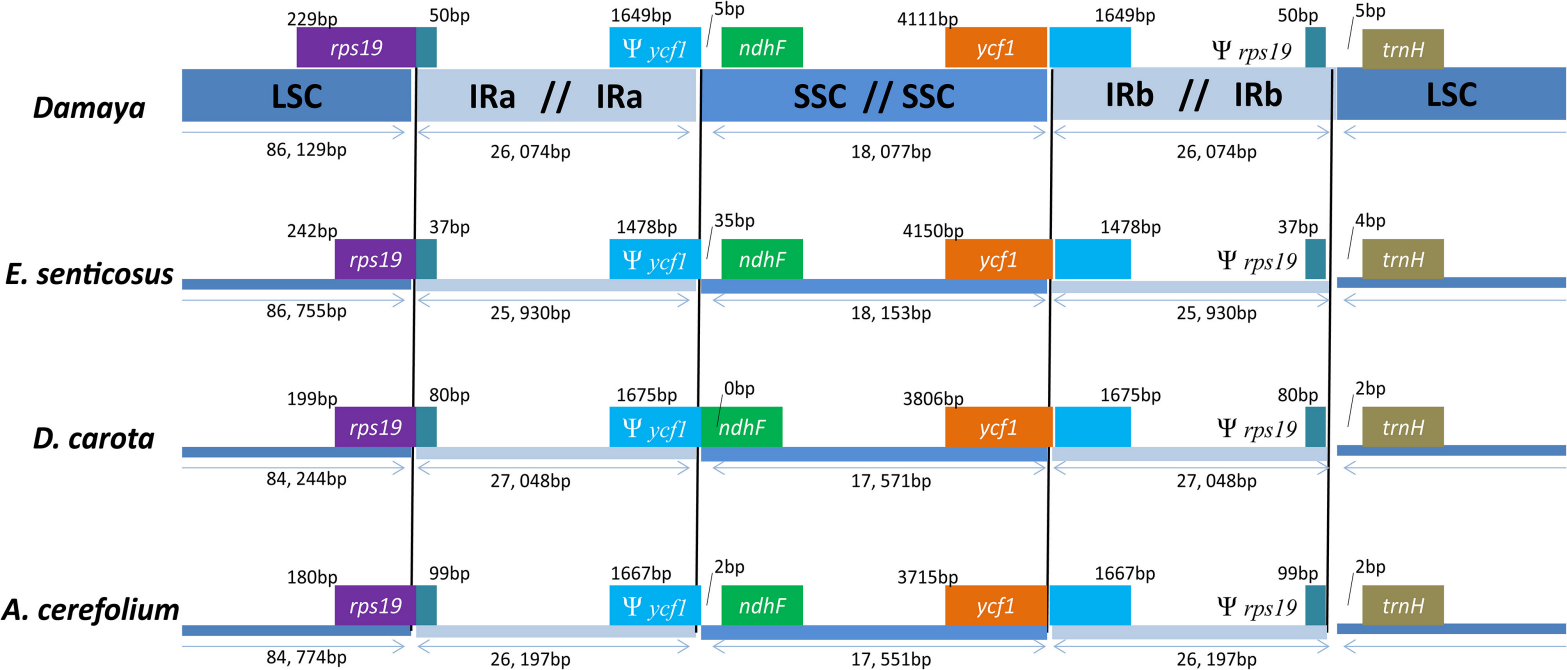
**A comparison of LSC, SSC, and IR region borders among four chloroplast genomes**.

The contraction and expansion at the borders of IR regions were common evolutionary events, which primarily contributed to the observed variation in the size of chloroplast genomes (Goulding et al., 1996; Wang et al., 2008). To elucidate this phenomenon and mechanism in Apiales, *P*. *ginseng* DMY chloroplast genome was used as the representative for comparison to the other three closely related chloroplast genomes, namely, those of *E. senticosus, A*. *cerefolium*, and *D*. *carota*. Detailed comparisons of LSC, SSC, and IR boundaries in these four representatives from Apiales were shown in Figure 3. The *rps19* and *ycf1* genes crossed the LSC/IRa and SSC/IRb boundaries, respectively. At the same time, the pseudogene fragment of *rps19* and *ycf1* (Ψ *rps19* and Ψ *ycf1*) were located at the IRb/LSC and IRa/SSC boundaries, respectively. In *P*. *ginseng* DMY, IRb carried a short Ψ *rps19* fragment with 50 bp at the IRb/SSC boundary, and IRa carried a short Ψ *ycf1* fragment with 1649 bp at the IRa/SSC boundary. Compared to Ψ *rps19*, the length of Ψ *ycf1* had a wider range of variation (1478–1675 bp). The Ψ *ycf1* fragment in *E*. *senticosus* is the shortest, and the length of Ψ *ycf1* in *P*. *ginseng* DMY, *A*. *cerefolium*, and *D*. *carota* is almost identical. Therefore, we may infer that the expansion and contraction of IR region in *P*. *ginseng* chloroplast genome is fairly stable compared to that of the other chloroplast genomes in Apiales.

#### Selection pressure on P. ginseng chloroplast genome

To explore the selection pressures of *P*. *ginseng* chloroplast genome, DMY chloroplast genome was compared to the other three chloroplast genomes in Apiales (*E*. *senticosus, A*. *cerefolium*, and *D. carota*; Table 4). Furthermore, to clarify the variations in each part of chloroplast genomes, the sequences of LSC, SSC, and IR regions were divided into three functional regions, including a protein-coding region (CDS), intron region, and intergenic spacers (IGS) region. The polymorphic site numbers between *P*. *ginseng* and *E*. *senticosus* (1721) were less than those between *P*. *ginseng* and *D*. *carota* (9916) or *A*. *cerefolium* (9504). Nucleotide differences between *P*. *ginseng* and *D*. *carota* or *A*. *cerefolium* were significantly higher than those between *P*. *ginseng* and *E*. *senticosus* for all three functional regions. Thus, we can infer that *P*. *ginseng* is more closely related to *E*. *senticosus* than the other two, which is also reflected in the phylogenetic trees (Figure 2 and Supplementary Figure S1).

**Table 4:** **Comparison of protein-coding region (CDS), intron, and intergenic spacers (IGS) at LSC, IR, and SSC regions of chloroplast genomes**.

		***P*. *ginseng*/*D*. *carota***					***P*. *ginseng*/*A*. *cerefolium***					***P*. *ginseng*/*E*. *senticosus***				
**Region**		**NP**	**ND**	**Ka**	**Ks**	**Ka/Ks (*P*-value)**	**NP**	**ND**	**Ka**	**Ks**	**Ka/Ks (*P*-value)**	**NP**	**ND**	**Ka**	**Ks**	**Ka/Ks (*P*-value)**
CDS	LSC	1958	0.0443	0.0212	0.1484	0.1427 (0)	2021	0.0458	0.0226	0.1496	0.1511 (0)	352	0.0079	0.004	0.0229	0.1765 (8.5e-58)
	IR	496	0.0258	0.0224	0.0407	0.5507 (8.3e-10)	470	0.0243	0.0221	0.0351	0.6276 (4.9e-6)	62	0.0032	0.0037	0.0015	2.528 (0.0142)
	SSC	1296	0.0907	0.0721	0.2321	0.3107 (1.9e-99)	1276	0.0895	0.0708	0.2314	0.306 (2e-100)	281	0.0193	0.0154	0.0391	0.3937 (1.7e-13)
	TOTAL	3750	0.0482	0.0305	0.1316	0.2319 (0)	3767	0.0485	0.031	0.1308	0.2371 (0)	695	0.0089	0.006	0.0202	0.2988 (7e-54)
Intron	LSC	713	0.0962	–	–	–	711	0.0962	–	–	–	106	0.0139	–	–	–
	IR	46	0.0174	–	–	–	30	0.0113	–	–	–	4	0.0015	–	–	–
	SSC	115	0.1136	–	–	–	115	0.1130	–	–	–	109	0.0282	–	–	–
	TOTAL	874	0.0789	–	–	–	856	0.0774	–	–	–	130	0.0115	–	–	–
IGS	LSC	3898	0.1295	–	–	–	3871	0.1260	–	–	–	719	0.0210	–	–	–
	IR	768	0.0284	–	–	–	413	0.0151	–	–	–	68	0.0025	–	–	–
	SSC	626	0.1797	–	–	–	597	0.1722	–	–	–	109	0.0282	–	–	–
	TOTAL	5292	0.0873	–	–	–	4881	0.0793	–	–	–	896	0.0137	–	–	–
TOTAL		9916	0.0664	–	–	–	9504	0.0632	–	–	–	1721	0.0111		–	–

*This is a summary table of each calculation from three different comparisons of P. ginseng vs. D. carota, P. ginseng vs. A. cerefolium, and P. ginseng vs. E. senticosus. Abbreviation: NP, the numbers of polymorphic sites; ND, nucleotide differences; Ks, synonymous substitution differences; and Ks, nonsysnonymous substitution differences. In the column Ka/Ks, there are two numbers, in which the above number is the Ka/Ks value and the below value in bracket is the p-value*.

From Table 4, it is apparent that SSC region has the highest variation ratio for all three functional regions, and IR region has the lowest variation ratio, which was the same as the chloroplast genome of *Sesamum indicum L* (Yi and Kim, 2012; Zhang et al., 2013). The Ka/Ks values of IR region were higher than those of LSC and SSC regions. Moreover, this trend was consistent with all comparison results of between *P*. *ginseng* and *D*. *carota* or *A. cerefolium*. In addition, the Ka/Ks value of CDS IR region between *P*. *ginseng* and *E*. *senticosus* was 2.528 (*p*-value = 0.0142), indicating a strong positive selection on the IR region. To further investigate the evolutionary rate in the IR region, the Ka/Ks values for all protein-coding gene in IR region between *P*. *ginseng* and *E*. *senticosus* were calculated using the KaKs_Calculator 2.0 with MA method. Among the six genes (*ndhB, rpl2, rpl23, rps7, ycf2*, and *ycf15*) in IR region, the Ka/Ks values for *ndhB, rpl2, rpl23*, and *rps7* were NA, the Ka/Ks value for *ycf2* was 2.38 (Ka = 0.0055, Ks = 0.0023, *p*-value = 0.124), and the Ka/Ks value for *ycf15* was 50 (Ka = 0.0039, Ks = 7.7e-5, *p*-value = 0). It's obvious that those exceptionally high Ka/Ks value were mainly caused by extremely low Ks value, which could not well reflect the real selection pressure on these genes. Even though, we could infer that *ycf2* and *ycf15* suffered positive selection. *ycf2* and *ycf15* have been predicted in many chloroplast genomes by several previous studies, and experimental evidences have proved that they are indeed functional (Drescher et al., 2000; Dong et al., 2013; Shi et al., 2013). Anja Drescher and et al have proved that *ycf2* played an essential role in cell survival in tobacco chloroplast (Drescher et al., 2000), and *ycf15* was also found transcribed but not spliced in spinach (Schmitz-Linneweber et al., 2001).

To make more comprehensive investigation on selection pressure of all chloroplast genes, we have calculated the Ka/Ks value for all chloroplast genes between DMY and the three closely related species (*E. senticosus, A. cerefolium*, and *D. carota*). From Supplementary Table S7, we found that, when comparing the genes between DMY and *E. senticosus*, many genes (such as *atpE, atpF* and so on) have high Ka/Ks values, which were also introduced by extremely low Ks value. When comparing DMY with the three closely related-species, besides these genes with low Ks introduced high Ka/Ks values, *ycf1, ycf2*, and *ycf15* indeed suffered positive selection with Ka/Ks value no less than 0.5, which were also found in tobacco and *Sedum sarmentosum* chloroplast genomes(Drescher et al., 2000; Dong et al., 2013). Furthermore, Kapralov MV and Filatov DA found that *rbcL* evolved under strong positive selection in *Schiedea* and plants from Amaranthaceae family(Kapralov and Filatov, 2006; Kapralov et al., 2012), while *rbcL* did not suffered obviously positive selection in *P. ginseng*, which indicates that gene may suffers different selection pressure in different plants.

### Polymorphism and phylogenetic analysis of ginseng chloroplast genome sequences within species

#### Comparisons of the five P. ginseng chloroplast genome sequences

Genetic variations in chloroplast genome sequences usually have significant value in population genetic analyses and plant domestications (Tang et al., 2004; Kawakami et al., 2007; Yang et al., 2013). To explore the evolution of *P*. *ginseng*, comparative analyses among the four newly sequenced chloroplast genomes and *P*. *schinseng* Nees chloroplast genome were performed. DMY, EMY, and GLS had identical chloroplast genome sequences, and the YSS chloroplast genome sequence had a 1 bp insertion located at base 5472 reference to DMY chloroplast genome sequence. We also compared the previously reported *P*. *schinseng* Nees chloroplast genome with *P*. *ginseng* DMY chloroplast genome. *P*. *schinseng* Nees chloroplast genome was sequenced by ABI 377 with an average coverage of 4.7×, and *P*. *ginseng* DMY chloroplast genome was sequenced by HiSeq2000 with an average coverage over 1000× and Roche/454 GS-FLX (Titanium) with an average coverage of 217×. Five sites on *P*. *schinseng* Nees chloroplast genome sequence (accession number in GenBank: NC_006290) were undefined, four were marked as R (purine, A or G) and one was Y (pyrimidine, T or C). All five sites were excluded from the differentiated sites, because those bases in DMY at the same site were also a purine or pyrimidine. Excluding the undefined sites, a total of 172 variant sites, including 88 indels and 84 substitutions, were found (Supplementary Table S8). According to the annotation of DMY chloroplast genome, 30 of 84 substitution sites were located in intergenic region, 16 of them were located in intron region, 2 were located in rRNA gene *rrn23*, 1 was located in tRNA gene *trnV-UAC*, and the remaining 35 were located in protein-coding genes (7 for *ycf1*; 5 for *rpoC2*; 4 for *accD* and *psbD*; 2 for *atpB* and *rpoB*; and 1 for *rps2, rps12, lhbA, ndhD, psaA, psaB, psaC, atpF, atpI, clpP*, and *atpA*). Regarding 88 indels, 51 were located in intergenic region, 35 were located in intron region, and 2 were located in tRNA genes (*trnC-GCA* and *trnS-UGA*). Further analyzing the relationship between repeat regions of these indel sites, the result showed that 21 sites were located in the short single nucleotide acid repeat regions, with a repeat unit occurrence of five or more times. Thus, the different origins of *P*. *schinseng* Nees and *P*. *ginseng* DMY could account for the differences in their chloroplast genome sequences, and other reasons require further investigation.

#### Minor allele site polymorphisms and evolution of P. ginseng chloroplast genome

**Figure 4:**
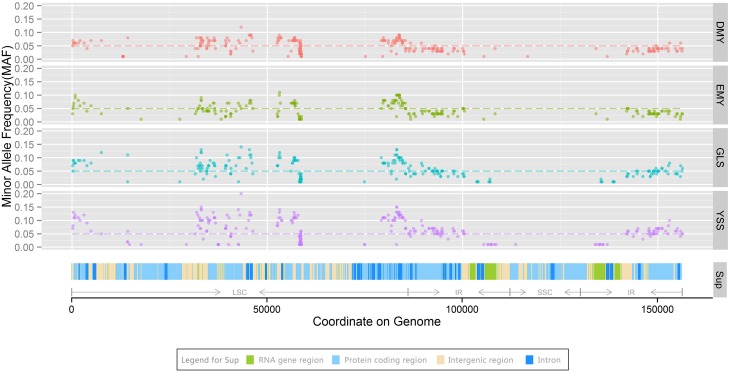
**Minor allele distribution among chloroplast genomes of four strains**. In the panels DMY, EMY, GLS, and YSS, the dashed lines indicate a minor allele frequency (MAF) of 0.05. The panel Sup shows tRNA and rRNA genes, protein-coding, intergenic, and intron regions on the chloroplast genome, the colors are shown in the legend for Sup. LSC, SSC, and IR regions are marked using arrows.

Minor allele frequency (MAF) refers to the frequency of the least common allele occurs in a given population and is usually used for conducting evolutionary analyses of genetic markers (Zhang et al., 2012a; McPherson et al., 2013). Diversity in the MAF has been widely employed for evolutionary and genetic analyses between domestic and wild species in both plants and animals (Vasemagi et al., 2012; Alhaddad et al., 2013; Iorizzo et al., 2013; Petersen et al., 2013). There are a large number of chloroplast genomes in per plant cell (Pyke, 1999). In current study, the DNA sample for each strain was extracted from numerous cells. This means that we took all these chloroplast genomes from the same individual as a chloroplast population for each strain. In the assembly section, the assembled genome sequences substantially represent major alleles in the population. To study the heterogeneity of chloroplast genomes and the dynamics of minor alleles over the course of *P*. *ginseng* domestication, all minor allele sites for DMY, EMY, GLS, and YSS were identified with high-resolution reads. After mapping the high-quality reads of all four strains in DMY chloroplast genome respectively and filtering minor alleles with an MAF value of <0.01 (Kolz et al., 2009; Liu et al., 2012), a total of 312 polymorphism sites were detected (Figure 4). To avoid interference from sequencing errors and DNA migration from chloroplast genome to mitochondria and nuclear genomes (Thorsness and Weber, 1996; Birky, 2001; Zhang et al., 2012b), and to restrict analyses to common alleles (De et al., 2008; Skoglund and Jakobsson, 2011; Iorizzo et al., 2013), minor alleles sites with an MAF value of ≥0.05 were used as credible minor alleles, and the cutoff line was shown as a dashed horizontal line in Figure 4. With this MAF value, 208 minor allele sites were identified from all four strains (Supplementary Table S9). And the major types in variation of all these common SNP sites were immobile in every strain. Excluding the variant between YSS and the other three strains at base 5472, there were 5 deletions (3 for A, 1 for C, and 1 for CG) and 202 substitutions, including 41 transitions (transitions of A–G occurred 11 times, all other types transitions occurred 10 times). Among 161 transversion events, the occurrences A–C and T–G accounted for the majority part, 68 and 52 times, respectively. 136 of 208 minor alleles were located in LSC region, and the other 72 were located in IR region. According to the annotation of DMY chloroplast genome, 52 were located in intergenic region, 35 were in intron region, 1 was in tRNA gene *trnD-GUC*, and the other 120 were in protein-coding region (73 non-synonymous and 47 synonymous substitutions). For the protein-coding genes in LSC region, the details for minor allele number in each gene were shown as follows: 13 in *rbcL*; 6 in *psaB*; 6 in *psbC*; 4 in *psbD, psaA, atpB*, and *rpl22*; 3 in *rps8*; 2 in *psbA, ycf3, petD, rpoA, rpl14*, and *rps3*; and 1 in *matK, infA*, and *rpl16* for each. For the protein-coding genes in IR region, the number of minor alleles would be doubled because there are two copies of IR (for each copy, 20 in *ycf2*, 4 in *rp12*, 3 in *ycf15* and *ndhB*, and 1 in *rps19* and *rpl23*).

**Figure 5:**
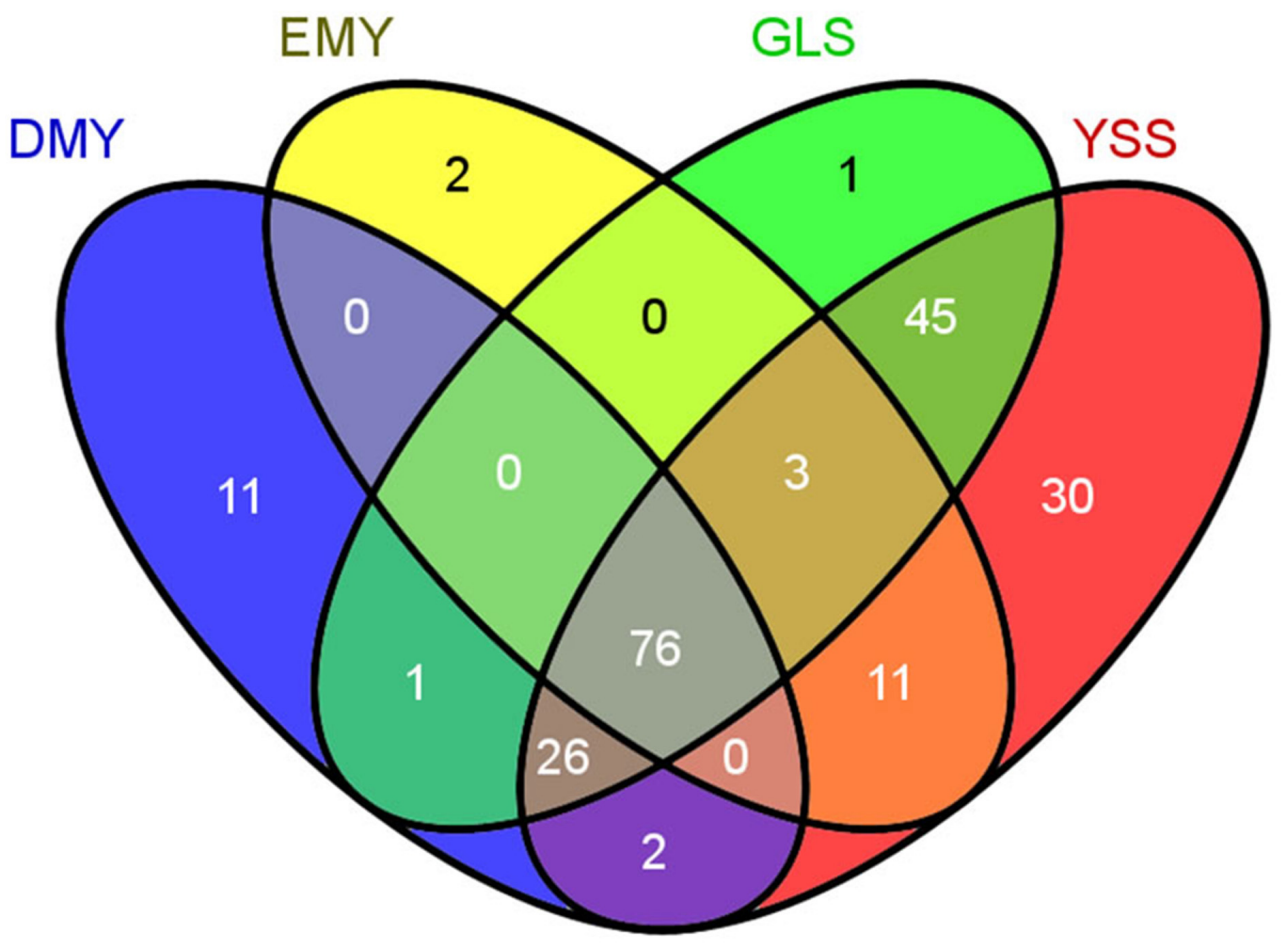
**Minor allele overlaps among chloroplast genomes of four strains**.

For all 208 chloroplast minor allele sites with MAF ≥0.05, DMY, EMY, GLS, and YSS covered 116, 92, 152, and 193, respectively. The polymorphism site numbers per kb of the chloroplast genome sequences for these four strains were 0.74, 0.59, 0.97, and 1.23, respectively. The minor allele overlaps among DMY, EMY, GLS, and YSS was 76 (Figure 5). All these 76 common minor allele sites were shared by all four strains; 29 of them were located in protein-coding region (14 non-synonymous and 15 synonymous substitutions). Additionally, there were 30 specific minor allele sites in YSS, with 23 in protein-coding region (15 non-synonymous and 8 synonymous substitutions). In DMY, there were also 11 specific minor allele sits, of which 6 were in *rbcL*. Those specific minor allele sites in YSS and DMY could be used as genetic markers to discriminate them from others. However, the specific minor allele sites in GLS and EMY were very few, so it was very difficult to choose applicable minor allele sites. When the threshold for MAF value was increased to 0.1, the total number of minor alleles decreased to 88, among which DMY, EMY, GLS, and YSS covered 1, 11, 35, and 86, respectively. Seen from the minor allele analysis result, the wild-type *P*. *ginseng* YSS is very different from the other three cultivated strains.

**Figure 6:**
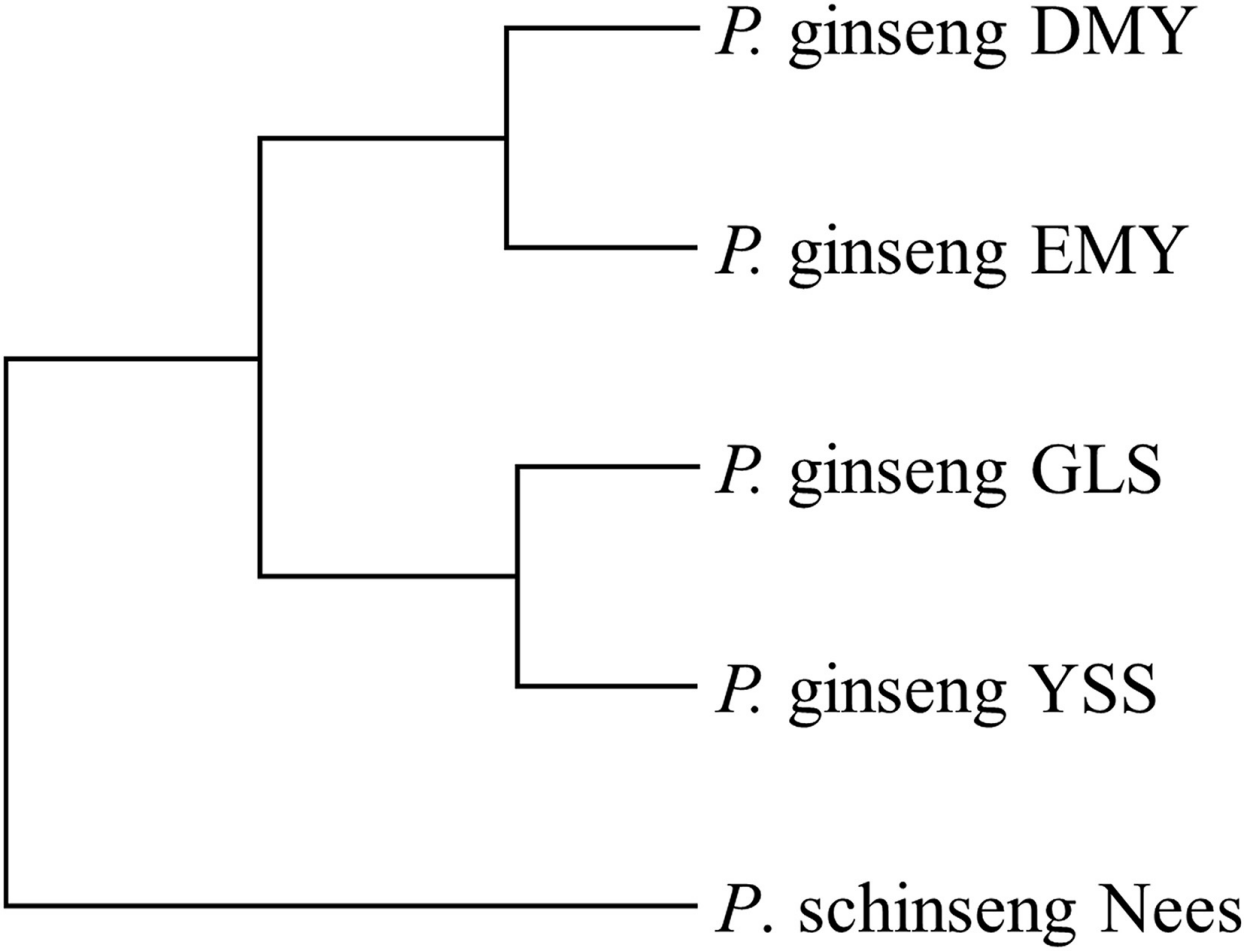
**The evolutionary relationship among five ginseng chloroplast genomes**.

At the same time, from Figure 4, we found that those minor alleles on chloroplast genomes were exposed to purifying selection due to domestication. Based on the information of minor allele sites and the differences of genome sequences, an evolutionary relationship among the four newly sequenced strains and the previously reported *P*. *schinseng* Nees can be inferred (Figure 6). Greater divergence in genome sequences was found between *P*. *schinseng* Nees and the four newly sequenced Chinese ginseng species; thus, *P*. *schinseng* Nees is an outgroup. Additionally, we can infer that DMY is closely related to EMY, while GLS is closely related to YSS.

## Author contributions

Yongbing Zhao, Jingfa Xiao, Xumin Wang, Xiaobo Qu and Jun Yu conceived and designed the experiments. Jinlong Yin, Haiyan Guo, Yongbing Zhao, Wen Xiao and Xiaobo Qu performed the experiments. Yongbing Zhao, Yuyu Zhang, Chen Sun, Jiayan Wu, Xumin Wang and Jingfa Xiao analyzed and interpreted the data. Haiyan Guo, Jinlong Yin and Xumin Wang contributed reagents/materials or analysis tools. Yongbing Zhao, Jinlong Yin, Haiyan Guo, Xumin Wang, Jingfa Xiao and Jun Yu wrote the manuscript. All authors contributed to and approved the final manuscript.

### Conflict of interest statement

The authors declare that the research was conducted in the absence of any commercial or financial relationships that could be construed as a potential conflict of interest.
