# Supplementary material for: The complete chloroplast genome provides insight into the evolution and polymorphism of Panax ginseng
Source: Front Plant Sci. 2015 Jan 14;5:696. doi: 10.3389/fpls.2014.00696 (PMC4294130; doi:10.3389/fpls.2014.00696)
Supplement: Supplementary file 1 [file Table1.DOC]

|  | YSS | DMY | EMY | GLS |
| --- | --- | --- | --- | --- |
| body | lumpish | cylindrical | cylindrical | cylindrical |
| body grains | tight and fine | rough and broken | rough and broken | rough and thin |
| rhizome | longer | short and thick | threadlike | short and thick |
| rhizome nodes | dense | few and big | few and small | few and big |
| lateral root | few | many | few | few |
| fibrous nodules | pearl and distinct | inconspicuous | inconspicuous | inconspicuous |

Supplementary Table S1: Morphological differences among four *Panax* giseng strains
