# Supplementary material for: The complete chloroplast genome provides insight into the evolution and polymorphism of Panax ginseng
Source: Front Plant Sci. 2015 Jan 14;5:696. doi: 10.3389/fpls.2014.00696 (PMC4294130; doi:10.3389/fpls.2014.00696)
Supplement: Supplementary file 2 [file Table2.DOC]

Supplementary Table S2: High quality reads number (pairs number) for four kinds of *Panax* ginseng strains

|  | **Illumina HiSeq2000** | | | | **Roche GS FLX** |
| --- | --- | --- | --- | --- | --- |
|  | 180bp | 500bp | 1~2k | 2kb |  |
| Damaya | / | 3,162,775 | 760,278 | / | 3,318,800 (1.7 GB) |
| Ermaya | 2,270,377 | 2,737,712 | / | / | / |
| Gaolishen | / | 1,729,226 | / | 1,641,652 | / |
| Yeshanshen | / | 2,424,098 | / | 1,303,565 | / |

In the above table, left slash means this kind reads were not available for this strain. In current study, all reads from Illumina Hiseq 2000 are pair-end with 100bp in length for single read, and the number in the above table are pairs number. The reads from Roche GS FLX are single end, and the number in above table is single reads number.
