# Supplementary material for: The complete chloroplast genome provides insight into the evolution and polymorphism of Panax ginseng
Source: Front Plant Sci. 2015 Jan 14;5:696. doi: 10.3389/fpls.2014.00696 (PMC4294130; doi:10.3389/fpls.2014.00696)
Supplement: Supplementary file 4 [file Table4.DOC]

Supplementary Table S4: Codon usage and the corresponding tRNA gene in DMY chloroplast genome

| **Amino Acid** | **Codon** | **No.*** | **Ratio** | **tRNA Gene** |  | **Amino Acid** | **Codon** | **No.*** | **Ratio** | **tRNA Gene** |
| --- | --- | --- | --- | --- | --- | --- | --- | --- | --- | --- |
| Ala | GCT | 626 | 0.023 |  |  | Leu | TTA | 837 | 0.031 | trnL-UAA |
|  | GCC | 227 | 0.008 |  |  |  | TTG | 580 | 0.022 | trnL-CAA*2 |
|  | GCA | 400 | 0.015 | trnA-UGC*2 |  |  | CTT | 585 | 0.022 |  |
|  | GCG | 156 | 0.005 |  |  |  | CTC | 187 | 0.007 |  |
| Arg | CGT | 341 | 0.013 | trnR-ACG*2 |  |  | CTA | 385 | 0.014 | trnL-UAG |
|  | CGC | 85 | 0.003 |  |  |  | CTG | 187 | 0.007 |  |
|  | CGA | 369 | 0.014 |  |  | Phe | TTT | 929 | 0.035 |  |
|  | CGG | 129 | 0.004 |  |  |  | TTC | 536 | 0.02 | trnF-GAA |
|  | AGA | 484 | 0.018 | trnR-UCU |  | Pro | CCT | 418 | 0.015 |  |
|  | AGG | 179 | 0.006 |  |  |  | CCC | 213 | 0.008 |  |
| Asn | AAT | 950 | 0.036 |  |  |  | CCA | 311 | 0.011 | trnP-UGG |
|  | AAC | 299 | 0.011 | trnN-GUU*2 |  |  | CCG | 164 | 0.006 |  |
| Asp | GAT | 852 | 0.032 |  |  | Ser | AGT | 413 | 0.015 |  |
|  | GAC | 222 | 0.008 | trnD-GUC |  |  | AGC | 119 | 0.004 | trnS-GCU |
| Cys | TGT | 216 | 0.008 |  |  |  | TCT | 568 | 0.021 |  |
|  | TGC | 76 | 0.002 | trnC-GCA |  |  | TCC | 328 | 0.012 | trnS-GGA |
| Gln | CAA | 694 | 0.026 | trnQ-UUG |  |  | TCA | 393 | 0.015 | trnS-UGA |
|  | CAG | 239 | 0.009 |  |  |  | TCG | 191 | 0.007 |  |
| Glu | GAA | 1040 | 0.039 | trnE-UUC |  | Thr | ACT | 537 | 0.02 |  |
|  | GAG | 341 | 0.013 |  |  |  | ACC | 257 | 0.009 | trnT-GGU |
| Gly | GGT | 595 | 0.022 |  |  |  | ACA | 402 | 0.015 | trnT-UGU |
|  | GGC | 188 | 0.007 |  |  |  | ACG | 161 | 0.006 |  |
|  | GGA | 696 | 0.026 | trnG-UCC |  | Trp | TGG | 459 | 0.017 | trnW-CCA |
|  | GGG | 325 | 0.012 |  |  | Tyr | TAT | 765 | 0.029 |  |
| His | CAT | 478 | 0.018 |  |  |  | TAC | 195 | 0.007 | trnY-GUA |
|  | CAC | 142 | 0.005 | trnH-GUG |  | Val | GTT | 510 | 0.019 |  |
| Ile | ATT | 1047 | 0.04 |  |  |  | GTC | 176 | 0.006 | trnV-GAC*2 |
|  | ATC | 469 | 0.017 | trnI-GAU*2 |  |  | GTA | 506 | 0.019 | trnV-UAC |
|  | ATA | 698 | 0.026 |  |  |  | GTG | 212 | 0.008 |  |
| Lys | AAA | 1017 | 0.038 | trnK-UUU |  | Ter | TGA | 23 | 0 |  |
|  | AAG | 366 | 0.013 |  |  |  | TAA | 41 | 0.001 |  |
| Met | ATG | 606 | 0.023 | trnM-CAU*2 |  |  | TAG | 22 | 0 |  |

The (×2) symbol after the tRNA gene name means two copies for that genes.
