# Supplementary material for: The complete chloroplast genome provides insight into the evolution and polymorphism of Panax ginseng
Source: Front Plant Sci. 2015 Jan 14;5:696. doi: 10.3389/fpls.2014.00696 (PMC4294130; doi:10.3389/fpls.2014.00696)
Supplement: Supplementary file 6 [file Table6.DOC]

Supplementary Table S6: genes utilized for phylogenetic analysis

| **Group of gene** | **Gene list** |
| --- | --- |
| Subunits of ATP synthase | *atpA, atpB, atpE, atpH, atpI* |
| Subunits of NADH-dehydrogenase | *ndhC, ndhD, ndhE, ndhG, ndhH, ndhI, ndhJ* |
| Subunits of cytochrome b/f complex | *petA, petG, petL, petN* |
| Subunits of photosystem I | *pasA, psaB, psaC, psaI, psaJ* |
| Subunits of photosystem II | *psbA, psbC, psbD, psbE, psbF, psbH, psbI, psbJ, psbK, psbL, psbM, psbN, psbT* |
| Large subunit of ribosome | *rpl14, rpl20, rpl22, rpl32, rpl33, rpl36* |
| Small subunit of ribosome | *rps2, rps4, rps7, rps8, rps11, rps14, rps15, rps18, rps19* |
| Maturase | *matK* |
| Large subunit of rubisco | *rbcL* |
| DNA dependent RNA polymerase | *rpoC2* |
