# Supplementary material for: The complete chloroplast genome provides insight into the evolution and polymorphism of Panax ginseng
Source: Front Plant Sci. 2015 Jan 14;5:696. doi: 10.3389/fpls.2014.00696 (PMC4294130; doi:10.3389/fpls.2014.00696)
Supplement: Supplementary file 10 [file Image1.PDF]

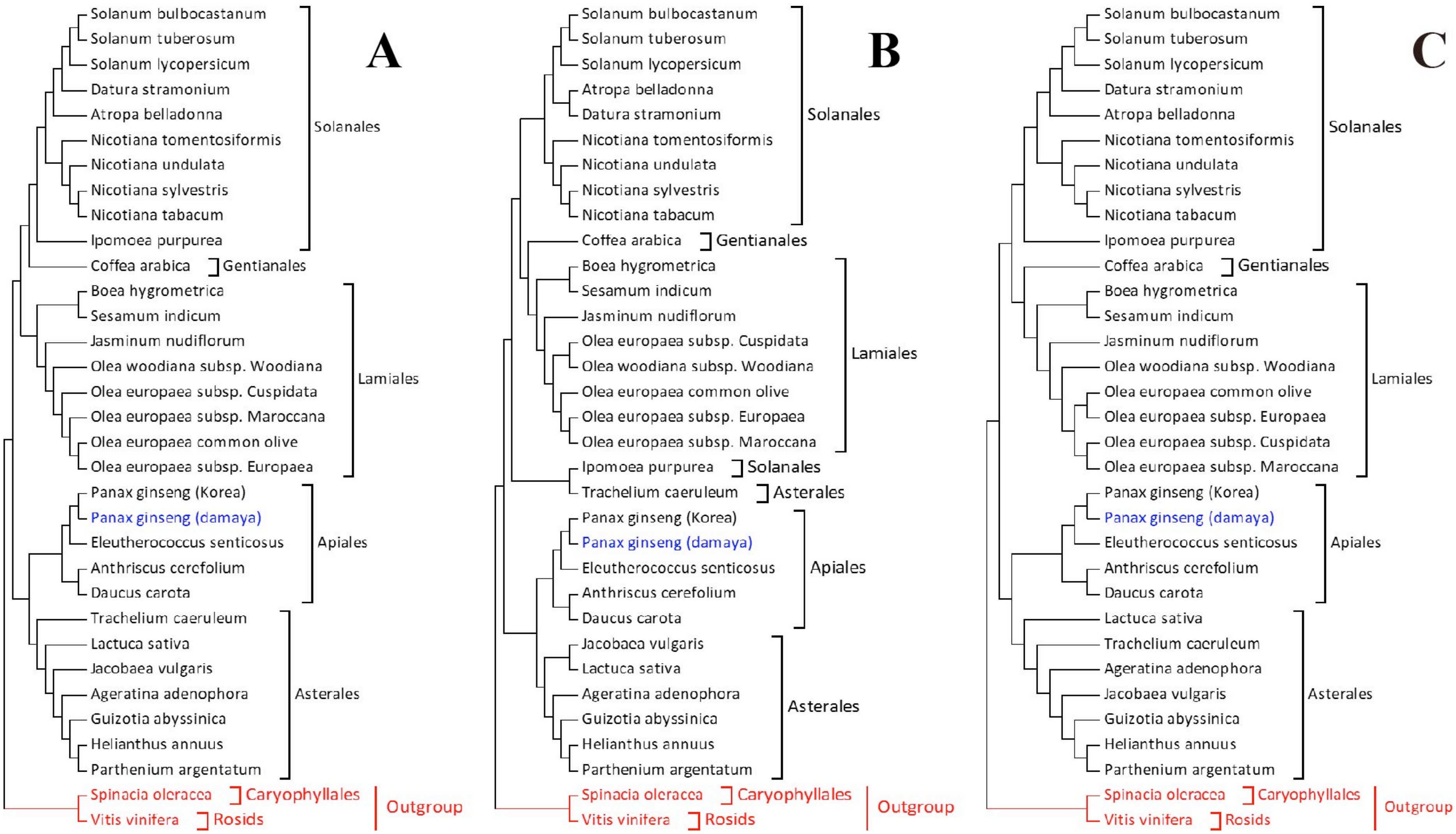
A
Solanum bulbocastanum
Solanum tuberosum
Solanum lycopersicum
Datura stramonium
Atropa belladonna
Nicotiana tomentosiformis
Nicotiana undulata
Nicotiana sylvestris
Nicotiana tabacum
Ipomoea purpurea
Solanales
Coffea arabica
Gentianales
Boea hygrometrica
Sesamum indicum
Jasminum nudiflorum
Olea woodiana subsp. Woodiana
Olea europaea subsp. Cuspidata
Olea europaea subsp. Maroccana
Olea europaea common olive
Olea europaea subsp. Europaea
Lamiales
Panax ginseng (Korea)
Panax ginseng (damaya)
Eleutherococcus senticosus
Anthriscus cerefolium
Daucus carota
Apiales
Trachelium caeruleum
Lactuca sativa
Jacobaea vulgaris
Ageratina adenophora
Guizotia abyssinica
Helianthus annuus
Parthenium argentatum
Asterales
Spinacia oleracea
Caryophyllales
Vitis vinifera
Rosids
Outgroup
B
Solanum bulbocastanum
Solanum tuberosum
Solanum lycopersicum
Atropa belladonna
Datura stramonium
Nicotiana tomentosiformis
Nicotiana undulata
Nicotiana sylvestris
Nicotiana tabacum
Solanales
Coffea arabica
Gentianales
Boea hygrometrica
Sesamum indicum
Jasminum nudiflorum
Olea europaea subsp. Cuspidata
Olea woodiana subsp. Woodiana
Olea europaea common olive
Olea europaea subsp. Europaea
Olea europaea subsp. Maroccana
Lamiales
Ipomoea purpurea
Solanales
Trachelium caeruleum
Asterales
Panax ginseng (Korea)
Panax ginseng (damaya)
Eleutherococcus senticosus
Anthriscus cerefolium
Daucus carota
Apiales
Jacobaea vulgaris
Lactuca sativa
Ageratina adenophora
Guizotia abyssinica
Helianthus annuus
Parthenium argentatum
Asterales
Spinacia oleracea
Caryophyllales
Vitis vinifera
Rosids
Outgroup
C
Solanum bulbocastanum
Solanum tuberosum
Solanum lycopersicum
Datura stramonium
Atropa belladonna
Nicotiana tomentosiformis
Nicotiana undulata
Nicotiana sylvestris
Nicotiana tabacum
Ipomoea purpurea
Solanales
Coffea arabica
Gentianales
Boea hygrometrica
Sesamum indicum
Jasminum nudiflorum
Olea woodiana subsp. Woodiana
Olea europaea common olive
Olea europaea subsp. Europaea
Olea europaea subsp. Cuspidata
Olea europaea subsp. Maroccana
Lamiales
Panax ginseng (Korea)
Panax ginseng (damaya)
Eleutherococcus senticosus
Anthriscus cerefolium
Daucus carota
Apiales
Lactuca sativa
Trachelium caeruleum
Ageratina adenophora
Jacobaea vulgaris
Guizotia abyssinica
Helianthus annuus
Parthenium argentatum
Asterales
Spinacia oleracea
Caryophyllales
Vitis vinifera
Rosids
Outgroup

Supplementary Figure S1. Phylogenetic tree based on the whole nucleotide sequences from three different regions of chloroplast genome (Maximum likelihood). Damaya was marked with blue color and two out-group species were marked with red color. A) The phylogenetic tree based on the sequence at the LSC region. B) The phylogenetic tree based on the sequence at the IR region. C) The phylogenetic tree based on the sequence at SSC region.
